# Respiratory syncytial virus prophylaxis for prevention of recurrent childhood wheeze and asthma: a protocol for a systematic review

**DOI:** 10.1186/s13643-019-1251-x

**Published:** 2019-12-19

**Authors:** Lauren Alexandra Quinn, Michael D. Shields, Helen E. Groves

**Affiliations:** 1 Queen’s University Belfast, Belfast, Northern Ireland; 2 Child Health, Centre for Experimental Medicine, Welcome Wolfson Institute for Experimental Medicine, Queen’s University Belfast, Belfast, Northern Ireland; 3 Queen’s University Belfast, Belfast, Northern Ireland

**Keywords:** Respiratory syncytial virus, Asthma, Recurrent wheeze, Prophylaxis, Monoclonal antibody, Immunoprophylaxis

## Abstract

**Background:**

Lower respiratory tract infection (LRTI) caused by respiratory syncytial virus (RSV) has been associated with greater risk of recurrent wheezing and subsequent asthma. However, it is still unclear whether this association is causal or not. RSV-specific monoclonal antibodies have been shown to reduce RSV-related hospitalisations in high-risk infants, i.e. those born pre-term, but the longer term follow-up has given conflicting evidence for the prevention of recurrent wheeze or asthma.

**Objective:**

We aim to perform a systematic review and meta-analysis to determine whether or not prophylaxis with a monoclonal antibody for prevention of RSV-bronchiolitis reduces the risk of subsequent recurrent wheeze or asthma. If so, this would support the hypothesis that the association between RSV and recurrent wheeze and/or asthma is causative.

**Methods:**

To identify relevant studies, we will search a number of databases including Medline, Embase, PubMed and Web of Science and will also manually look for unpublished data by contacting the manufacturers of monoclonal antibodies. The intervention being investigated is RSV-specific monoclonal antibody prophylaxis, and the outcome being measured is recurrent wheeze and/or asthma. Studies will be screened according to inclusion/exclusion criteria, to include primary studies of any study design type. Eligible studies will then be evaluated for quality and assessed for bias independently by three reviewers using the ‘Grading of Recommendations Assessment, Development and Evaluation’ (GRADE) approach. The results of the studies will be extracted into 2 × 2 outcome tables, and a meta-analysis will be carried out to produce forest plots based on relative risk. Heterogeneity will be assessed using the *I*^2^ statistic. The statistical software we will use is StatsDirect.

**Discussion:**

This review will aid in determining if the relationship between RSV and asthma development is a causal one, by showing the effect (if any) of RSV prophylaxis on subsequent recurrent wheeze/asthma. If this study shows RSV prophylaxis to have no effect on the outcome of recurrent wheeze/asthma, the question of causality remains.

## Background

Acute bronchiolitis is an extremely common acute lower respiratory tract infection in infants, with symptoms including coughing, shortness of breath, crackles, wheezing and poor nutrition [1]. The majority of young children will experience bronchiolitis, and approximately 3% will require hospital admission [1]. In many countries, bronchiolitis is the most common reason for hospitalisation of young children [1]. It has been shown that lower respiratory tract infections in early life, particularly in infancy, are associated with development of recurrent wheeze and asthma in later childhood [2]. Pre-term infants especially are at an increased risk of both severe bronchiolitis and recurrent wheeze or asthma development independently [3, 4].

The pathogenesis of asthma is multifactorial, but in simple terms, asthma causes hypersensitivity and inflammation of the airways, with common symptoms being wheeze and shortness of breath [5]. Recurrent wheeze in infancy has a significant effect on the quality of life of both the patients and their families [6]. An international study was carried out which surveyed random samples of the general population of infants. They found that 45.2% of infants in the study population had at least one wheezing episode, and 20.3% had recurrent wheeze, defined as three or more episodes of wheezing [7]. Asthma is the most prevalent chronic respiratory disease worldwide [8]. It has been estimated that the cost of asthma is approximately £1.1 billion in the UK, highlighting it as a key public health issue [9]. It creates a huge burden on both patients and health services in terms of quality of life and cost, with the most significant impact being amongst lower socioeconomic groups and ethnic minorities [10]. With the overall prevalence increasing globally, further research is needed into why this increase is happening, and whether or not there are any preventative measures that can be undertaken [10].

Acute bronchiolitis in early life is very strongly associated with an increased risk of asthma development [11]. It has been shown that infants hospitalised with acute bronchiolitis have a significantly increased risk of developing childhood wheeze and asthma, with one study from Finland finding the risk of recurrent wheeze or asthma development, after acute bronchiolitis at less than 6 months, to be twice that of the general population [12]. However, while this association is very well established, the mechanism by which this may occur is poorly understood; thus, this relationship is yet to be proven as being causal [13]. There is much debate over whether or not acute bronchiolitis is merely just the first manifestation of asthma, rather than being the cause of it. To assess causality, studies assessing the prevention of the proposed risk factor, i.e. bronchiolitis, on the outcome of asthma are needed [11].

The most common cause of acute bronchiolitis is respiratory syncytial virus (RSV), most often in infants up to 12 months. Rhinovirus is also a cause of acute bronchiolitis, generally occurring in slightly older infants [12]. RSV is an RNA virus which uses surface glycoproteins G and F to infect cells [14]. RSV-specific monoclonal antibodies are drugs which have shown efficacy in reducing RSV hospitalisations in high-risk infants, such as those born prematurely [15]. They work by binding to this F glycoprotein, preventing viral invasion of the host cells. This provides passive immunity by blocking the fusion of infected cells and reducing cell-to-cell transmission and viral activity [14, 16]. These monoclonal antibodies have a half-life of approximately 3 weeks hence the need for once-monthly injections during RSV season, to maintain a prophylactic level [17].

The main example of RSV-specific monoclonal antibodies is palivizumab. Palivizumab is a humanised monoclonal antibody which has been found to be effective in reducing hospitalisations due to RSV bronchiolitis in high-risk infants. It is injected once monthly from November to March as this is the typical RSV season [15]. Palivizumab has been repeatedly proven to be safe and well tolerated with very low rates of minor adverse events such as injection site reaction, fever, diarrhoea and irritability [18].

Motavizumab is derived from palivizumab, therefore making it a second-generation humanised monoclonal antibody. It was originally thought to display better efficacy and therefore had a lower dose requirement when compared to palivizumab [18, 19]. However, it is important to note that motavizumab was discontinued in 2010 due to questions due to its side effect profile, particularly in regard to serious skin reactions, and questions over whether or not it was actually more efficacious than palivizumab [18, 20, 21].

Other RSV-specific monoclonal antibody biosimilars to palivizumab do exist. Suptavumab was developed recently; however, it failed to meet its primary endpoint in clinical trials and was withdrawn in 2017 [22]. Even more recently developed is nirsevimab, which has a longer half-life than palivizumab thus offers protection against RSV through one single intramuscular injection [23]. Lunamab is another RSV-specific monoclonal antibody which was developed as a cheaper biosimilar to palivizumab aimed at low-income countries [24]. However, given that these are only recently developed, it is unlikely we will come across any longer term follow-up studies with regard to recurrent wheeze.

Monoclonal antibodies are expensive drugs. It is estimated that the cost of palivizumab is around £3000–£5000 per child [25]. Despite its proven efficacy and the high prevalence of RSV infection in infancy, most children will not experience a severe illness; therefore, it is not cost-effective to give to all infants [13, 25]. A systematic review analysing the cost-effectiveness of RSV prophylaxis based on the outcome of bronchiolitis found that it is cost-effective within certain subgroups of infants who are considered to be at high risk. These subgroups include very early pre-term infants (< 32 weeks), children with congenital heart disease and aboriginal children [26]. It also found that in infants of 33–35 weeks gestational age, RSV prophylaxis could be cost-effective against bronchiolitis if also based on the presence of certain risk factors which include chronological age, number of siblings, history of atopy, absence of breast-feeding, cigarette smoke exposure and day care attendance [27].

While these cost-effectiveness analyses have concluded that passive immunoprophylaxis is not financially viable for all infants born late pre-term (33–35 weeks), they have mainly been based on the outcome of RSV bronchiolitis itself, and not recurrent wheeze [28]. Given that the lungs of infants born late pre-term are not as immunologically developed as those born over 35 weeks, and also given the fact that the RSV hospitalisation rate amongst these late pre-term infants ranges between 3.75 and 9.8%, it is clear that this is a population which cannot be ignored [29]. A sub-group analysis in this gestational age group of infants will highlight their relative risk of recurrent wheeze after receiving RSV-specific monoclonal antibody prophylaxis and potentially re-open the discussion on the cost-effectiveness of monoclonal antibodies in this sub-group of pre-term infants.

The aim of this systematic review is to determine whether or not giving monoclonal antibody RSV prophylaxis in infancy reduces the risk of recurrent wheeze or asthma development in later childhood. This will then potentially provide some answers to the question of causality in the association of RSV infection and subsequent asthma.

## Methods/design

This systematic review and meta-analysis will be investigating if intervention with RSV prophylaxis compared with no prophylaxis has any effect on the outcome of recurrent wheeze or asthma in a population of infants born early pre-term to term. A literature search will be carried out across a number of databases, including Medline, Embase, Web of Science and PubMed, using a comprehensive search strategy. We will also contact the manufacturers of the monoclonal antibodies for any unpublished data. Studies will then be screened according to title and abstract, and then text body using clear inclusion and exclusion criteria. This protocol follows the Preferred Reporting Items for Systematic Review and Meta-Analysis Protocols (PRISMA-P) recommendations, and the Preferred Reporting Items for Systematic Review and Meta-Analysis (PRISMA) standards will be adhered to in reporting the findings (Additional file 1: Appendix 1).

### Inclusion and exclusion criteria

Table 1 summarises the inclusion/exclusion criteria which will be used in study screening.

**Table 1:** Inclusion and Exclusion criteria: A table outlining the inclusion and exclusion criteria used when screening first by title and abstract and then by full text. Papers will be included if they are primary studies of any study design. The population being studied has to be infants born early pre-term up to term. The studies have to be investigating monoclonal antibody prophylaxis compared with no prophylaxis or placebo, on the outcome of recurrent wheeze or asthma. No studies investigating a population of infants with congenital defects will be included, and no other RSV prophylaxis or treatment apart from monoclonal antibody will be considered

Include:	Exclude:
All study designs	Reviews
Primary studies, including peer-reviewed and grey literature	Letters
All ethnicities	Not about prophylaxis
Population: infants born early pre-term up to term, followed up for 1–10 years	Population: infants with congenital heart defects
Intervention: RSV prophylaxis with monoclonal antibody	Any other interventions such as RSV prophylaxis or treatment with RSV-specific immune globulins, steroids, vaccines, macrolides etc.
Comparison: no prophylaxis or placebo	Comparison: different dosing regimen of monoclonal antibody
Outcome: recurrent wheeze or asthma development	Bronchiolitis caused by other allergens or viruses such as rhinovirus

### Types of studies and participants

This review will include all types of primary study design including randomised control trials, prospective observational case-control studies and cohort studies. All participants will be infants born early pre-term up to term and followed up from infancy to childhood (1–10 years) in line with the inclusion criteria.

### Intervention, comparison and outcome

The intervention being investigated is RSV-specific monoclonal antibodies for immunoprophylaxis. This is being compared against no RSV prophylaxis. The outcome being measured is development of subsequent recurrent wheeze and/or asthma.

### Information sources and search strategy

The literature search will be carried out electronically using a strategy developed in collaboration with the Queen’s University Belfast Medical Librarian. To ensure all potential literature is included, we will search Embase, Medline, PubMed, Web of Science and the Cochrane Library. We will also contact the manufacturers of the RSV-specific monoclonal antibodies for any unpublished data and search trial registries such as ‘ClinicalTrials.gov’ and ‘BMC Trials’ for potentially suitable studies that may be imminently reported. An example of the planned electronic search strategy including limits applied can be seen in Table 2.

**Table 2:** Search strategy. An example of the comprehensive literature search which will be carried out across the electronic databases, with search terms and limitations applied. This search strategy example is from Embase

#	Searches	Results
1	Respiratory Syncytial Virus Infections/	1279
2	“RSV infection*”.mp. [mp=title, abstract, heading word, drug trade name, original title, device manufacturer, drug manufacturer, device trade name, keyword, floating subheading word, candidate term word]	4504
3	Asthma/	211,704
4	“asthma development*”.mp. [mp=title, abstract, heading word, drug trade name, original title, device manufacturer, drug manufacturer, device trade name, keyword, floating subheading word, candidate term word]	1070
5	wheez*.mp. [mp=title, abstract, heading word, drug trade name, original title, device manufacturer, drug manufacturer, device trade name, keyword, floating subheading word, candidate term word]	30,231
6	Respiratory Hypersensitivity/	3915
7	atopy.mp. [mp=title, abstract, heading word, drug trade name, original title, device manufacturer, drug manufacturer, device trade name, keyword, floating subheading word, candidate term word]	26,375
8	1 or 2	5266
9	3 or 4 or 5 or 6 or 7	244,215
10	8 and 9	976
11	limit 10 to english language	913
12	limit 11 to “all child (0 to 18 years)” [Limit not valid in Embase; records were retained]	913
13	limit 12 to journal article [Limit not valid in Embase; records were retained]	913
14	(later or subsequent*).mp. [mp=title, abstract, heading word, drug trade name, original title, device manufacturer, drug manufacturer, device trade name, keyword, floating subheading word, candidate term word]	1,504,151
15	risk factors/	537,637
16	“clinical factor*”.mp. [mp=title, abstract, heading word, drug trade name, original title, device manufacturer, drug manufacturer, device trade name, keyword, floating subheading word, candidate term word]	23,741
17	14 or 15 or 16	2,032,400
18	13 and 17	345
19	prophylaxis.mp.	205,494
20	Primary Prevention/	37,244
21	monoclonal antibody.mp. or Antibodies, Monoclonal/	261,479
22	palivizumab.mp. or Palivizumab/	2796
23	motavizumab.mp.	252
24	prevention.mp.	1,698,423
25	19 or 20 or 24	1,786,756
26	21 or 22 or 23	263,326
27	25 and 26	17,965
28	18 and 27	62

### Data collection and analysis

#### Selection of studies

The studies will be independently screened according to the inclusion and exclusion criteria by two reviewers. The screening will be a two-step process, first by title and abstract, and then by full-text, with those excluded by full text listed and explained in the appendix of the final report. A third-party reviewer will be involved in the case of any disagreements. Using a reference software (Mendeley), any duplicate articles will be identified. Any relevant reviews found in the literature search will only be used to source additional primary studies for this review.

#### Data extraction and management

We will use the standard Population, Intervention, Comparison and Outcome (PICO) approach. The population is defined as infants born at less than 36 weeks. The intervention is monoclonal antibody prophylaxis, compared to a placebo/no monoclonal antibody prophylaxis, and the primary outcome is recurrent wheeze and asthma development. Data will be extracted using an adapted form of the ‘Data collection form for Intervention review – RCTs and non-RCTs’ of the Cochrane Collaboration [30]. An example of this is in Additional file 1: Appendix 2. Data will be presented in a summary of findings table including the types of studies, population number, number in intervention, comparison groups, 2 × 2 outcome results tables, relative risk and a column for evaluation of the quality of evidence and bias risk. This summary of findings table will be presented in the results section, as per the Cochrane handbook [31]. If any data is missing, we will contact the authors of the paper to obtain the complete set.

#### Risk of bias

The risk of bias and quality of evidence will be evaluated independently by three reviewers using the ‘Grading of Recommendations Assessment, Development and Evaluation’ (GRADE) approach. This grades the evidence as being of high, moderate, low or very low quality by using the study design as a starting point and upgrading or downgrading the evidence according to certain criteria. Five factors which lower the quality of the evidence include limitations of study design and execution leading to bias, inconsistency or heterogeneity, indirectness, imprecision and publication bias [32]. The most likely bias to occur is sponsorship bias as a lot of the studies are likely to be funded by the manufacturers of the monoclonal antibodies.

#### Outcomes

To ensure comparability between studies, the primary outcome being investigated for this review is any recurrent wheeze or asthma, including parent-reported wheeze as well as formally doctor-diagnosed wheeze or asthma. Parent-reported wheeze is an important outcome to include as not all infants who wheeze will be assessed by a physician. It is possible that individual studies may measure other relevant outcomes such as RSV hospitalisation or allergy diagnosis; however, these are not a priority for this review.

#### Data synthesis and meta-analysis

Using the main outcome of recurrent wheeze (dichotomous—yes/no) and the data from the 2 × 2 outcome tables produced, a meta-analysis will be performed using a random-effects model, with relative risk as the principal summary measure. Individual studies will be represented on a forest plot based on relative risk and 95% confidence intervals. Funnel plots will also be generated to portray publication bias or possible selective reporting within studies. The software which will be used for the meta-analysis is StatsDirect statistical software [33]. Sub-group analysis will be carried out in infants born 33–35 weeks gestational age to compare their relative risk of recurrent wheeze after receiving RSV-specific monoclonal antibodies with those born at an earlier gestational age. This will aim to provide insight as to whether it may be worth considering monoclonal antibody prophylaxis against RSV for infants in this age group.

#### Heterogeneity

To test for heterogeneity (inconsistency between studies), we will use the *I*^2^ test, taking an *I*^2^ of > 75% as being high heterogeneity. Sub-group analysis looking particularly at late pre-term infants may be carried out to explore the effectiveness of RSV prophylaxis on subsequent recurrent wheeze in this population.

#### Safety

Rates of adverse events such as injection site reactions/allergic reactions, fever, and rash in both intervention and control groups will be extracted and compared to evaluate the safety of the intervention.

## Discussion

Asthma is the most prevalent chronic respiratory disease worldwide creating a huge burden on patients and services [7]. If this review demonstrates that RSV prophylaxis reduces asthma risk, this supports the hypothesis of a causal relationship between RSV infection and asthma development. This could have potentially huge clinical implications if there is the possibility of reducing rates of recurrent childhood wheeze with the use of RSV-specific monoclonal antibodies. This result and the subsequent sub-group analysis could also have significant implications in terms of which infants qualify as being ‘at-risk’ enough to receive the monoclonal antibodies and could thereafter open the discussion and allow for future studies on cost-effectiveness analysis of monoclonal antibodies with regard to reduction of recurrent wheeze. Also, if the review supports the hypothesis of a causal relationship between RSV bronchiolitis and subsequent recurrent wheeze, this will prompt further studies on the biological mechanism by which this may occur. On the contrary, if RSV prophylaxis is shown to have no effect on rates of asthma development, this will then open the debate further into whether the association between RSV infection in infancy and subsequent asthma development is actually causal or not.

## Supplementary information

**Additional file 1.** **Appendix 1.** PRISMA-P 2015 Checklist. Preferred reporting items for systematic review and meta-analysis protocols (PRISMA-P) 2015 checklist. **Appendix 2:** Data collection form for Intervention review – RCTs and non-RCTs. Adapted from the Cochrane Collaboration.

## Data Availability

Not applicable

## Abbreviations

GRADE: ‘Grading of Recommendations Assessment, Development and Evaluation’
RSV: Respiratory syncytial virus
