# Supplementary material for: Respiratory syncytial virus prophylaxis for prevention of recurrent childhood wheeze and asthma: a protocol for a systematic review
Source: Syst Rev. 2019 Dec 19;8:333. doi: 10.1186/s13643-019-1251-x (PMC6924058; doi:10.1186/s13643-019-1251-x)
Supplement: Supplementary file 1 — Additional file 1. Appendix 1. PRISMA-P 2015 Checklist. Preferred reporting items for systematic review and meta-analysis protocols (PRISMA-P) 2015 checklist. Appendix 2: Data collection form for Intervention review – RCTs and non-RCTs. Adapted from the Cochrane Collaboration. [file 13643_2019_1251_MOESM1_ESM.docx]

**Appendix:**

| **Section/topic** | **#** | **Checklist item** | **Information reported** | | **Line number(s)** |
| --- | --- | --- | --- | --- | --- |
|  |  |  | **Yes** | **No** |  |
| **ADMINISTRATIVE INFORMATION** | | | | | |
| **Title** | | | | | |
| Identification | 1a | Identify the report as a protocol of a systematic review | √ |  | 1 - 2 |
| Update | 1b | If the protocol is for an update of a previous systematic review, identify as such |  | √ |  |
| **Registration** | 2 | If registered, provide the name of the registry (e.g., PROSPERO) and registration number in the Abstract | √ |  |  |
| **Authors** | | | | | |
| Contact | 3a | Provide name, institutional affiliation, and e-mail address of all protocol authors; provide physical mailing address of corresponding author | √ |  | 8 - 15 |
| Contributions | 3b | Describe contributions of protocol authors and identify the guarantor of the review | √ |  | 332 - 335 |
| **Amendments** | 4 | If the protocol represents an amendment of a previously completed or published protocol, identify as such and list changes; otherwise, state plan for documenting important protocol amendments |  | √ |  |
| **Support** | | | | | |
| Sources | 5a | Indicate sources of financial or other support for the review |  | √ | 329 - 330 |
| Sponsor | 5b | Provide name for the review funder and/or sponsor |  | √ |  |
| Role of sponsor/funder | 5c | Describe roles of funder(s), sponsor(s), and/or institution(s), if any, in developing the protocol |  | √ |  |
| **INTRODUCTION** | | | | | |
| **Rationale** | 6 | Describe the rationale for the review in the context of what is already known | √ |  | 77-167 |
| **Objectives** | 7 | Provide an explicit statement of the question(s) the review will address with reference to participants, interventions, comparators, and outcomes (PICO) | √ |  | 169 - 172 |
| **METHODS** | | | | | |
| **Eligibility criteria** | 8 | Specify the study characteristics (e.g., PICO, study design, setting, time frame) and report characteristics (e.g., years considered, language, publication status) to be used as criteria for eligibility for the review | √ |  | 190 – 208 |
| **Information sources** | 9 | Describe all intended information sources (e.g., electronic databases, contact with study authors, trial registers, or other grey literature sources) with planned dates of coverage | √ |  | 209 - 216 |
| **Search strategy** | 10 | Present draft of search strategy to be used for at least one electronic database, including planned limits, such that it could be repeated | √ |  | 216 - 218 |
| ***STUDY RECORDS*** | | | | | |
| Data management | 11a | Describe the mechanism(s) that will be used to manage records and data throughout the review | √ |  | 229 - 236 |
| Selection process | 11b | State the process that will be used for selecting studies (e.g., two independent reviewers) through each phase of the review (i.e., screening, eligibility, and inclusion in meta-analysis) | √ |  | 221 - 227 |
| Data collection process | 11c | Describe planned method of extracting data from reports (e.g., piloting forms, done independently, in duplicate), any processes for obtaining and confirming data from investigators | √ |  | 233 – 235  462 - 522 |
| **Data items** | 12 | List and define all variables for which data will be sought (e.g., PICO items, funding sources), any pre-planned data assumptions and simplifications | √ |  | 229 - 240 |
| **Outcomes and prioritization** | 13 | List and define all outcomes for which data will be sought, including prioritization of main and additional outcomes, with rationale | √ |  | 252 = 258 |
| **Risk of bias in individual studies** | 14 | Describe anticipated methods for assessing risk of bias of individual studies, including whether this will be done at the outcome or study level, or both; state how this information will be used in data synthesis | √ |  | 242 - 250 |
| ***DATA*** | | | | | |
| **Synthesis** | 15a | Describe criteria under which study data will be quantitatively synthesized | √ |  | 262 - 266 |
|  | 15b | If data are appropriate for quantitative synthesis, describe planned summary measures, methods of handling data, and methods of combining data from studies, including any planned exploration of consistency (e.g., *I* ^2^, Kendall’s tau) | √ |  | 262 - 278 |
|  | 15c | Describe any proposed additional analyses (e.g., sensitivity or subgroup analyses, meta-regression) | √ |  | 268 - 272 |
|  | 15d | If quantitative synthesis is not appropriate, describe the type of summary planned |  | √ |  |
| **Meta-bias(es)** | 16 | Specify any planned assessment of meta-bias(es) (e.g., publication bias across studies, selective reporting within studies) | √ |  | 266 - 267 |
| **Confidence in cumulative evidence** | 17 | Describe how the strength of the body of evidence will be assessed (e.g., GRADE) | √ |  | 243 - 248 |

Appendix 1.

**PRISMA-P 2015 Checklist.** Preferred reporting items for systematic review and meta-analysis protocols (PRISMA-P) 2015 checklist.

**Data Extraction Form:**

# General Information

| Date form completed *(dd/mm/yyyy)* |  |
| --- | --- |
| Name/ID of person extracting data |  |
| Reference citation |  |
| Study author contact details |  |
| Publication type *(e.g. full report, abstract, letter)* |  |
| Notes: | |

# Study eligibility

| Study Characteristics | Eligibility criteria  *(Insert inclusion criteria for each characteristic as defined in the Protocol)* | | Eligibility criteria met? | | | Location in text or source *(pg & ¶/fig/table/other)* |
| --- | --- | --- | --- | --- | --- | --- |
|  |  |  | Yes | No | Unclear |  |
| Type of study | Randomised Controlled Trial | |  |  |  |  |
|  | Quasi-randomised Controlled Trial | |  |  |  |  |
|  | Controlled Before and After Study  Contemporaneous data collection  Comparable control sites  At least 2 x intervention and 2 x control clusters | |  |  |  |  |
|  | Interrupted Time Series  At least 3 time points before and 3 after the intervention  Clearly defined intervention point | |  |  |  |  |
|  | Other design (specify): | |  |  |  |  |
| Participants |  | |  |  |  |  |
| Types of intervention |  | |  |  |  |  |
| Types of comparison |  | |  |  |  |  |
| Types of outcome measures |  | |  |  |  |  |
| INCLUDE | | EXCLUDE | | | | |
| Reason for exclusion |  | | | | | |
| Notes: | | | | | | |

**DO NOT PROCEED IF STUDY EXCLUDED FROM REVIEW**

# Characteristics of included studies

## Methods

|  | **Descriptions as stated in report/paper** | | **Location in text or source** *(pg & ¶/fig/table/other)* |
| --- | --- | --- | --- |
| **Aim of study** *(e.g. efficacy, equivalence, pragmatic)* |  | |  |
| **Design** *(e.g. parallel, crossover, non-RCT)* |  | |  |
| **Unit of allocation** *(by individuals, cluster/ groups or body parts)* |  | |  |
| **Start date** |  | |  |
| **End date** |  | |  |
| **Duration of participation** *(from recruitment to last follow-up)* |  | |  |
| **Ethical approval needed/ obtained for study** | Yes No Unclear |  |  |
| **Notes:** | | | |

## Participants

|  | Description  *Include comparative information for each intervention or comparison group if available* | | Location in text or source *(pg & ¶/fig/table/other)* |
| --- | --- | --- | --- |
| Population description *(from which study participants are drawn)* |  | |  |
| Setting *(including location and social context)* |  | |  |
| Inclusion criteria |  | |  |
| Exclusion criteria |  | |  |
| Method of recruitment of participants *(e.g. phone, mail, clinic patients)* |  | |  |
| Informed consent obtained | Yes No Unclear |  |  |
| Total no. randomised *(or total pop. at start of study for NRCTs)* |  | |  |
| Clusters *(if applicable, no., type, no. people per cluster)* |  | |  |
| Baseline imbalances |  | |  |
| Withdrawals and exclusions *(if not provided below by outcome)* |  | |  |
| Age |  | |  |
| Sex |  | |  |
| Race/Ethnicity |  | |  |
| Severity of illness |  | |  |
| Co-morbidities |  | |  |
| Other relevant sociodemographics |  | |  |
| Subgroups measure |  | |  |
| Subgroups reported |  | |  |
| Notes: | | | |

## Intervention groups

|  | Description as stated in report/paper | Location in text or source *(pg & ¶/fig/table/other)* |
| --- | --- | --- |
| Group name |  |  |
| No. randomised to group *(specify whether no. people or clusters)* |  |  |
| Theoretical basis *(include key references)* |  |  |
| Description *(include sufficient detail for replication, e.g. content, dose, components)* |  |  |
| Duration of treatment period |  |  |
| Timing *(e.g. frequency, duration of each episode)* |  |  |
| Delivery *(e.g. mechanism, medium, intensity, fidelity)* |  |  |
| Providers *(e.g. no., profession, training, ethnicity etc. if relevant)* |  |  |
| Co-interventions |  |  |
| Economic information *(i.e. intervention cost, changes in other costs as result of intervention)* |  |  |
| Resource requirements *(e.g. staff numbers, cold chain, equipment)* |  |  |
| Integrity of delivery |  |  |
| Compliance |  |  |
| Notes: | | |

## Outcomes

|  | Description as stated in report/paper | | Location in text or source *(pg & ¶/fig/table/other)* |
| --- | --- | --- | --- |
| Outcome name |  | |  |
| Time points measured *(specify whether from start or end of intervention)* |  | |  |
| Time points reported |  | |  |
| Outcome definition *(with diagnostic criteria if relevant)* |  | |  |
| Person measuring/ reporting |  | |  |
| Unit of measurement *(if relevant)* |  | |  |
| Scales: upper and lower limits *(indicate whether high or low score is good)* |  | |  |
| Is outcome/tool validated? | Yes No Unclear |  |  |
| Imputation of missing data *(e.g. assumptions made for ITT analysis)* |  | |  |
| Assumed risk estimate *(e.g. baseline or population risk noted in Background)* |  | |  |
| Power *(e.g. power & sample size calculation, level of power achieved)* |  | |  |
| Notes: | | | |

## Other

| Study funding sources *(including role of funders)* |  |  |
| --- | --- | --- |
| Possible conflicts of interest *(for study authors)* |  |  |
| Notes: | | |

# Risk of Bias assessment

| Domain | Risk of bias | | | Support for judgement  *(include direct quotes where available with explanatory comments)* | Location in text or source *(pg & ¶/fig/table/other)* |
| --- | --- | --- | --- | --- | --- |
|  | Low | High | Unclear |  |  |
| Random sequence generation *(selection bias)* |  |  |  |  |  |
| Allocation concealment *(selection bias)* |  |  |  |  |  |
| Blinding of participants and personnel *(performance bias)* |  |  |  | Outcome group: All/ |  |
| *(if separate judgement by outcome(s) required)* |  |  |  | Outcome group: |  |
| Blinding of outcome assessment *(detection bias)* |  |  |  | Outcome group: All/ |  |
| *(if separate judgement by outcome(s) required)* |  |  |  | Outcome group: |  |
| Incomplete outcome data *(attrition bias)* |  |  |  | Outcome group: All/ |  |
| *(if separate judgement by outcome(s) required)* |  |  |  | Outcome group: |  |
| Selective outcome reporting? *(reporting bias)* |  |  |  |  |  |
| Other bias |  |  |  |  |  |
| Notes: | | | | | |

# Data and analysis

***For RCT/CCT***

***Dichotomous outcome***

|  | Description as stated in report/paper | | | | | Location in text or source *(pg & ¶/fig/table/other)* |
| --- | --- | --- | --- | --- | --- | --- |
| Comparison |  | | | | |  |
| Outcome |  | | | | |  |
| Subgroup |  | | | | |  |
| Time point *(specify from start or end of intervention)* |  | | | | |  |
| Results | Intervention | | | Comparison | |  |
|  | No. with event | Total in group | | No. with event | Total in group |  |
|  |  |  | |  |  |  |
| Any other results reported *(e.g. odds ratio, risk difference, CI or P value)* |  | | | | |  |
| No. missing participants |  | | |  | |  |
| Reasons missing |  | | |  | |  |
| No. participants moved from other group |  | | |  | |  |
| Reasons moved |  | | |  | |  |
| Unit of analysis *(by individuals, cluster/groups or body parts)* |  | | | | |  |
| Statistical methods used and appropriateness of these *(e.g. adjustment for correlation)* |  | | | | |  |
| Reanalysis required? *(specify, e.g. correlation adjustment)* | Yes No Unclear | |  | | |  |
| Reanalysis possible? | Yes No Unclear | |  | | |  |
| Reanalysed results |  | | | | |  |
| Notes: | | | | | | |

# Other information

|  | **Description as stated in report/paper** | **Location in text or source** *(pg & ¶/fig/table/other)* |
| --- | --- | --- |
| **Key conclusions of study authors** |  |  |
| **References to other relevant studies** |  |  |
| **Correspondence required for further study information** *(from whom, what and when)* |  | |
| **Notes:** | | |

Appendix 2:

**Data collection form for Intervention review – RCTs and non-RCTs**. Adapted from the Cochrane Collaboration.
